# Bottlenecks in the Efficient Use of Advanced Therapy Medicinal Products Based on Mesenchymal Stromal Cells

**DOI:** 10.1155/2015/895714

**Published:** 2015-07-27

**Authors:** Natalia Escacena, Elena Quesada-Hernández, Vivian Capilla-Gonzalez, Bernat Soria, Abdelkrim Hmadcha

**Affiliations:** ^1^Department of Stem Cells, Andalusian Centre for Molecular Biology and Regenerative Medicine (CABIMER), 41092 Seville, Spain; ^2^Newbiotechnic (NBT), Bollullos de la Mitación, 41110 Seville, Spain; ^3^CIBER de Diabetes y Enfermedades Metabólicas Asociadas (CIBERDEM), 08036 Barcelona, Spain

## Abstract

Mesenchymal stromal cells (MSCs) have been established as promising candidate sources of universal donor cells for cell therapy due to their contributions to tissue and organ homeostasis, repair, and support by self-renewal and multidifferentiation, as well as by their anti-inflammatory, antiproliferative, immunomodulatory, trophic, and proangiogenic properties. Various diseases have been treated by MSCs in animal models. Additionally, hundreds of clinical trials related to the potential benefits of MSCs are in progress. However, although all MSCs are considered suitable to exert these functions, dissimilarities have been found among MSCs derived from different tissues. The same levels of efficacy and desired outcomes have not always been achieved in the diverse studies that have been performed thus far. Moreover, autologous MSCs can be affected by the disease status of patients, compromising their use. Therefore, collecting information regarding the characteristics of MSCs obtained from different sources and the influence of the host (patient) medical conditions on MSCs is important for assuring the safety and efficacy of cell-based therapies. This review provides relevant information regarding factors to consider for the clinical application of MSCs.

## 1. Introduction

MSCs are considered a heterogeneous population of nonhaematopoietic progenitor cells derived from the mesodermal germ layer that have both self-renewal and multidifferentiation [1] abilities. MSCs found in virtually all postnatal organs and tissues [2] possess multifaceted features, making them promising candidate sources of donor cells for use in cell therapy and transplantation. MSCs function in the repair and support of tissues, contributing to tissue homeostasis. Although the exact origin of MSCs remains elusive, strong evidence has indicated that MSC progenitors are in the perivascular zone [3] in an environment that promotes a quiescent-resting state, ensuring homeostasis maintenance. When a tissue is damaged and the whole machinery of the organism begins to operate the body's repair mechanisms, MSCs enter the blood stream and are attracted by proinflammatory cytokines at injury areas. Thus, MSCs have been called “*guardians of inflammation*” [4]. The cytoskeleton, extracellular matrix molecules, cell contacts, adhesion ligands, and receptors are involved in the repair process [5]. Although the exact mechanisms related to the migration of MSCs into specific sites and across the endothelial cell layer remain unknown, chemokines and their receptors may play roles in this process.

Although MSC survival, permanent engraftment, and differentiation into resident cells was thought to be necessary to obtain the beneficial effects of these cells initially, clinical experience and several experiments have shown that one of the primary functions of MSCs, most likely their key function, is to secrete several bioactive molecules related to the microenvironment in which these cells are immersed. MSCs secrete a wide variety of proinflammatory and anti-inflammatory cytokines, chemokines, growth factors, and prostaglandins under resting and inflammatory conditions [6]. These molecules are associated with immunomodulation (indoleamine-2,3-dioxygenase (IDO), prostaglandin-E2 (PGE-2), TGF-*β*, HLA-G5, and HGF), antiapoptosis (VEGF, GM-CSF, TGF-*β*, Stanniocalcin-1, and IGF-I), angiogenesis (VEGF, MCP-1, and IGF-I), local stem and progenitor cell growth and differentiation support (SCF, Angiopoietin-1, and SDF-1), antifibrosis (HGF and bFGF), and chemoattraction (CCL2, CCL4, and CXCL12) [7]. Additionally, beneficial effects of the use of MSC conditioned media (CM) have been reported; even CM has been shown to be therapeutically better than the cells themselves [8, 9].

However, although these properties are generally attributed to all MSCs derived from different tissues, evidence from different studies has suggested that MSCs from diverse sources are not identical and do not always achieve the same efficacy levels and desired outcomes. Likewise, diverse donor conditions can affect the MSC characteristics because the environment “*niche*” in which MSCs are immersed may be affected. In this review, we will describe some of the biological characteristics of MSCs that must be considered and the effects of the disease status of donors and recipients on these characteristics.

## 2. Biological Characteristics

### 2.1. Phenotypic Profile

Since Friedenstein and colleagues first isolated a colony-forming unit fibroblast (CFU-F) from bone marrow (BM) [10], bone marrow has been widely used as a source of MSCs for many investigations and clinical trials. In addition to bone marrow, MSCs have been isolated from different tissues such as adipose tissue [11], umbilical cord blood [12], dental pulp [13], synovial liquid and amniotic fluid [14, 15]. All these tissues vary in their cellular components, signals, and factors secreted, resulting in different immediate microenvironment conditions, thus developing several physiological niches. Although isolated and long-term cultured MSCs of most tissues show similar immunophenotypic characteristics, some differences have been found among MSCs of different tissue origins according to data obtained by* in vitro* experiments. In 2006, the International Society of Cellular Therapy (ISCT) published the minimal criteria to define MSCs by nomenclature (Table 1) and by biological characteristics [10, 16–22] to allow studies from different groups to be compared and contrasted. These criteria include the following: (i) coexpression of markers such as CD73, CD90, and CD105 and a lack of expression of haematopoietic markers (CD45, CD34, and CD14) and human leucocyte antigen (HLA-DR), (ii) multipotent differentiation potential, and (iii) adherence to plastic. However, several researchers have noted that adipose-tissue-derived MSCs (AD-MSCs) express CD34 and CD54 in early passages [23] and have lower expression of CD106 and that umbilical cord blood-derived MSCs (UCB-MSCs) express CD90 and CD105 [24]. Other markers have been used in different studies, and other differences have emerged, such as VEGFR-2 (Flk-1) expression, which was significantly higher in periosteum-derived cells compared to that in adipose tissue- and muscle-derived cells, or the rate of NGFR positivity, which was much higher in muscle-derived cells compared to that in other mesenchymal tissue-derived cells [25].

Although some immunophenotypic differences have been documented, many researchers consider the fact that these differences could be due to distinct extraction methods and different culture methodologies, resulting in variations of MSC surface markers. Thus, this review aimed to further investigate markers and characteristics that are more specific to select the better sources of MSCs for clinical applications.

Likewise, expanding the cells* in vitro* is necessary to obtain the desired numbers for therapeutic approaches. Changes in the proteomic phenotype of AD-MSCs have been observed during passages [26], although no proper approaches to examine the state of cells continuously during long-term* in vitro* culture have been established. Some researchers ascribe these variations to the adaptation of cells to the environment; thus, determining the biomolecular markers that are involved in these variations is essential for obtaining a better phenotypic characterisation of these cells and thus for achieving more effective cell therapy in the future.

### 2.2. MSC Proliferation

The proliferative activity of MSCs is another feature that may be affected by the different origins of MSCs. The rate and persistence of MSC proliferation appear to vary between source tissues. MSCs are considered adult stem cells, and, unlike embryonic stem cells (ESCs), these cells have a limited proliferative capacity. Physiological niches maintain adult stem cells in an undifferentiated state; however, when MSCs are cultured* in vitro*, they age, which affects their therapeutic properties, such as alterations in phenotype, differentiation potential, global gene expression patterns, miRNA profiles [27], and even chromosomal abnormalities [28], particularly after long-term culture or when cells of multiple doublings are used. Large numbers of MSCs are needed for therapeutic applications, and* in vitro* expansion is required to produce the desired MSC numbers.* In vivo*, MSCs represent 0.0001% of nucleated BM cells, and their number decreases with the age of the donor. The quantity of MSCs (CFU-Fs) among nucleated BM cells decreases with age from one MSC in 10^4^ BM cells in newborns to one MSC in 10^5^ cells in teenagers and to one MSC in 10^6^ cells in older individuals [29]. Furthermore, MSCs from older human donors differ significantly from those from younger donors in morphology, replicative lifespan [30], doubling time, healing capacity [31], and differentiation potential. Sufficient evidence has indicated that MSCs from older donors have limited therapeutic efficacy, and some studies have suggested that the difference between preclinical and clinical findings is due to the donor age. Therefore, considering that several age-related diseases exist and that elderly patients are potential users of cell therapy, understanding the molecular and biological effects of ageing on MSCs is essential for developing safe and effective MSC-based autologous cell therapy. Meanwhile, the use of allogeneic MSCs may be a treatment option for these specific patients. As we comment below, MSCs elude allogeneic rejection, and their infusion is feasible and well tolerated, with no adverse effects [32, 33].

### 2.3. Differentiation Capacity

MSCs have the ability to differentiate* in vitro* into several mesenchymal lineages including adipose tissue, bone, cartilage, and muscle [15, 34, 35]. Furthermore, MSCs can differentiate into endothelial cells, neurons, and glial cells because MSCs express genes related to specific lineages rather than to those of the mesenchymal lineage [36]. Although multilineage differentiation is another minimal criterion advised by the ISCT and undoubtedly represents a fundamental property of MSCs, this ability depends primarily on the source tissue from which these cells are derived. As discussed by Sakaguchi et al. [25], who compared human MSCs isolated from bone marrow, synovium, periosteum, skeletal muscle, and adipose tissue and expanded them by similar processes, synovium-derived cells have the greatest ability for chondrogenesis; adipose- and synovium-derived cells have the greatest ability for adipogenesis; and bone marrow-, synovium-, and periosteum-derived cells have the greatest ability for osteogenesis. In another comparative analysis, UCB-MSCs showed no adipogenic differentiation capacity in contrast to BM- and AT-MSCs [37]. As discussed by Horwitz et al. [38], who used differentiated MSCs in a study to test the regeneration of damaged tissues, BM-MSCs can engraft after transplantation, differentiate to functional osteoblasts and contribute to the formation of new dense bone in children with osteogenesis imperfecta. Most likely, the microenvironment in which MSCs are transplanted directly influences their distinct differentiation pathways. New insights into the biological characteristics of MSCs are needed to achieve future therapies.

### 2.4. Immunomodulatory Actions

Immunomodulatory properties of MSCs and their immunoprivileged condition make these cells good candidates for use in several clinical trials related to chronic, inflammatory, and autoimmune diseases. MSCs interact with cells of the innate or adaptive immune system (T cells, B cells, NK cells, monocyte-derived dendritic cells, and neutrophils) [39, 40]. For a cell to be recognised by the immune system, the expression of major histocompatibility complex (MHC) and costimulatory molecules is necessary. MHC class I and class II human leukocyte antigens (HLAs) are master triggers of robust immunological rejection of grafts because they present antigens to cytolytic T lymphocytes (CTL) [41]. Human mesenchymal stem cells (hMSCs) are characterised by low expression of MHC class I HLAs but are constitutively negative for class II HLCs; these cells do not express costimulatory molecules such as B7-1, B7-2, CD80, CD86, CD40, and CD40L [42]. However, similar to the thymic epithelium, MSCs express the surface markers VCAM-1, ICAM-2, and LFA-3 [42, 43], which are crucial for T cell interactions. Although a T cell response should be expected, hMSCs are able to modulate the activation and proliferation of both CD4+ and CD8+ cells* in vitro* by arresting T cells in G0/G1 phase [44, 45]. Different studies have suggested that cell-cell interactions and certain soluble factors are the mechanisms used by MSCs to mediate the immune response. Factors such as IDO, TGF-*β*1, IFN-*γ*, IL-1*β*, TNF*α*, IL-6, IL-10, PGE-2, HGF, and HLA-G5 are secreted by MSCs or released after interactions with target cells. As we mentioned above, MSCs remain in a resting state, display antiapoptotic properties and maintain different cells such as haematopoietic stem cells (HSCs), thus contributing to tissue homeostasis. However, in an inflammatory environment such as that created by cytokines such as IFN-*γ*, TNF-*α*, IL-1*α*, and IL-1*β*, MSCs begin to exert their immunosuppressive effects and polarise, inhibiting the proliferation of effector cells and their production of cytokines. In this regard, IFN-*γ* is postulated as a “*licensing*” agent for MSC antiproliferative action. MSCs may also acquire behaviour as antigen-presenting cells (APCs) under certain concentrations of IFN-*γ* [46, 47]. However, no consensus regarding what concentration of IFN-*γ* is more necessary for MSCs to show that inhibitory or APC functions exists. Likewise, TNF-*α* is another proinflammatory cytokine involved in the MSC immune response, and TNF-*α* enhances the effect of IFN-*γ* [48]. IFN-*γ*, with or without the help of TNF-*α*, stimulates the production of IDO by MSCs, inhibiting the proliferation of activated T or NK cells [49] and thus enhancing the homing potential and reparative properties of these cells; however, some potential risks are associated with the role of IFN-*γ* [50].

Some authors have maintained that the immunomodulatory properties of MSCs are comparable [51, 52], while others have argued that MSCs of different tissue origins or species cannot have equivalent immunomodulatory properties [53, 54]. For example, MSCs from perinatal sources (umbilical cord and amniotic membrane) show a higher immunomodulatory capacity, differential gene expression profiles, and paracrine factor secretion compared to BM-MSCs [55]. Interestingly, in 2012, Lee and colleagues found that HLA-G, a specific MHC-I antigen that is critical for maintaining the immune-tolerant state of pregnancy and that is a contributing factor to the induction of stronger immunosuppression [56], is strongly positive only in placenta-derived MSCs (PD-MSCs) in contrast to BM-MSCs and AD-MSCs, suggesting that the immunophenotype of PD-MSCs may be superior to other MSCs in terms of their immunosuppressive function [57]. Nevertheless, in another related study, BM-MSCs were more immunomodulatory than PD-MSCs [58]. Melief et al. [59] concluded that the immunomodulatory capacities of BM-MSCs and AD-MSCs are similar but that differences in cytokine secretion cause AD-MSCs to have more potent immunomodulatory effects than BM-MSCs.

A 2002 study showed that allogeneic MSCs prolonged skin graft survival in baboons [60]. Mouse MSCs have been used in related experiments; these cells use inducible nitric oxide synthase (iNOS) for immunosuppression instead of IDO. These findings indicate that MSCs differ between species [61]. Since then, several preclinical models have been used to analyse the biological effects of MSCs and their ability to modulate immune responses, considering that not all animal models mimic human diseases.

Once more, these differences could be due to isolation procedures, to culture methodology, or, more likely, to differences in the microenvironments where cells reside. These and other findings lead us to believe that determining whether these differences may be relevant for their clinical applications and whether MSCs of a particular tissue type are more appropriate for specific therapies or diseases is important.

## 3. Preclinical Applications

Preclinical models are essential for clinicians, researchers, and both national and international regulatory agencies to demonstrate the safety and efficacy of MSC-based therapies [62]. Because MSCs are able to exert immunomodulatory properties and to act on different immune cells both* in vitro* and* in vivo* as mentioned above, these cells have begun to be used against autoimmune diseases based on various autoimmune experimental models. Pioneer studies in experimental autoimmune encephalomyelitis (EAE), a model for multiple sclerosis, reported that MSCs derived from various tissue origins show efficacy against neurodegenerative disorders [63–68]. BM-MSC and UCB-MSC treatments have brought about improvements in clinical and laboratory parameters in systemic lupus erythematosus (SLE) [33, 69]. Furthermore, ameliorating effects have been observed in experimental mouse models of rheumatoid arthritis (RA) [70]. Diabetes is another autoimmune disorder in which MSCs have been employed [71–73]. Although promising results and progress have been observed in this field, the interspecies differences and contradictory experimental outcomes, as well as the inability to recreate the complete pathophysiology of some diseases, make it necessary to search for new animal models to yield comparable results.

## 4. Autoimmune Diseases

MSCs are being used to facilitate the engraftment of transplanted HSCs and to treat graft-versus-host disease (GVHD) after allogeneic haematopoietic stem cell transplantation (HSCT) based on their immunomodulatory properties and their ability to provide appropriate conditions; however, preclinical and clinical experiments with MSCs do not always show similar results for the prevention and treatment of GVHD. In a study using a mouse model of GVHD [74], MSCs suppressed alloantigen-induced T cell proliferation* in vitro* in a dose-dependent manner but yielded no clinical benefit regarding the incidence or severity of GVHD. Instead, when UCB-MSCs were administered in weekly doses in a xenogeneic model of GVHD, a marked decrease in human T cell proliferation was observed, and none of the mice developed GVHD. No therapeutic effect was obtained when UCB-MSCs were administered at the onset of GVHD [75]. In the same line of research, serial infusions of mouse AD-MSCs could efficiently control the lethal GVHD that occurred in recipients transplanted with haploidentical haematopoietic grafts [76]. Mixed results have also been achieved in human patients. One study found that the cotransplantation of culture-expanded MSCs and HSCs from HLA-identical sibling donors after myeloablative therapy accelerated haematopoietic engraftment [77]; however, a significant reduction of GVHD symptoms was not shown, although the incidence or severity of GVHD did not increase. Koç et al. [78] reported a positive impact of MSCs on haematopoiesis; rapid haematopoietic recovery was observed in a clinical study with breast cancer patients who received autologous HSCT together with autologous MSCs. Therapeutic effects have also been reported at the onset of GVHD, such as the case of a 9-year-old boy with severe treatment-resistant GVHD after allogeneic HSCT for acute lymphocytic leukaemia who received haploidentical MSCs derived from his mother. He showed improvement after 2 MSC administrations [79]. Similar results have been obtained in steroid-refractory GVHD pilot studies with BM-MSCs and AD-MSCs [80, 81]. Several infusions appear to be required to maintain the level of active immunomodulation by MSCs. Similarly, the expression of proinflammatory cytokines such as IFN-*γ* in the environment at the time of MSC administration is required by these cells to exert their immunosuppressive effect because a lack of MSC “*licensing*” can result in the absence of the desired therapeutic effect.

While evidence that MSCs are effective in combination or after HSCT in specific haematological and nonhaematological diseases has been shown, adverse reactions and risk factors intrinsic to this practice have been reported. In a pilot study, HLA-identical sibling-matched HSCs were transplanted with or without MSCs in haematological malignancy patients. Although MSCs were well tolerated and this treatment effectively prevented GVHD, six patients (60%) in the MSC group and three (20%) in the non-MSC group had 3-year disease-free survival rates of 30 and 66.7%, respectively [82]. The relapse rate in the experimental group was higher than that in the control group, suggesting that MSCs may impair the therapeutic graft-versus-leukaemia (GVL) effect.* In vitro* and* in vivo* studies regarding the relationship between the immunosuppressive properties of MSCs and the stimulation of cancer growth have been performed. Mouse MSCs from BM, spleen, and thymus that were injected together with a genetically modified tumour cell vaccine could equally prevent the onset of an antitumour memory immune response, thus leading to tumour growth in normally resistant mice [83]. In another* in vivo* experiment with a murine melanoma tumour model, the authors observed that the subcutaneous injection of B16 melanoma cells led to tumour growth in allogeneic recipients only when MSCs were coinjected [84]. The functions of MSCs can be influenced by the existing microenvironment, making them acquire supportive properties towards cancer cells and decrease immune reactions [85]. Therefore, potential risks, related to the growth support and enhancement of undetected or “*resident*” cancer, do exist, and the administration of MSCs in these patients must be thoroughly evaluated.

## 5. Do MSCs Carry out the Patient's Disease?

One of the strategies to obtain MSCs for therapeutic purposes is an autologous approach. These cells are collected from patients by more or less invasive methods, isolated, seeded in culture under good manufacturing practice (GMP) quality standards, and reinjected into the patient. Nevertheless, when the repair mechanisms of the body are insufficient or ineffective, this treatment results in a homeostatic imbalance in the organism, producing degradation and disease and compromising the pool of endogenous cells, thus resulting in low efficacy. Some diseases provoke changes in the bone marrow microenvironment, which is one of the primary sources of MSCs, thus producing changes in the endogenous pool of MSCs and altering their biological features [86]. MSCs from patients with acute myeloid leukaemia showed abnormal biological properties, including morphological heterogeneity, limited proliferation capacity, and impaired differentiation and haematopoiesis support ability [87]. MSCs derived from patients with multiple myeloma showed impaired immune-inhibitory effects on T cells, decreasing their osteogenic potential [88]. Poor proliferation, differentiation potentials, and cytokine release defects were found in BM-MSCs derived from patients with aplastic anaemia, another haematopoietic disorder [89, 90].

Although the mechanisms remain unknown, MSCs appear to be involved in autoimmune pathologies. For instance, MSCs derived from patients with autoimmune diseases display the following altered functions. (i) MSCs from rheumatoid arthritis (RA) patients have an impaired ability to support haematopoiesis [91] and lower proliferative and clonogenic potentials [92]. (ii) MSCs from immune thrombocytopenic purpura (ITP) patients have a reduced proliferative capacity and a lower inhibitory effect on T cell proliferation compared with MSCs from healthy donors [93]. (iii) MSCs from systemic lupus erythematosus (SLE) patients display deficient growth, abnormal morphology, and upregulated telomerase activity [94, 95]. (iv) MSCs from systemic sclerosis (SSc) patients display early senescence [96]. In metabolic diseases such as diabetes, alterations in autologous MSCs have also been documented. A study using MSCs from type 2 diabetic mice showed that the number of these cells was diminished and that their proliferation and survival abilities were impaired* in vitro*. Moreover, diabetic MSC engraftment produced limited improvement in the diabetic subjects and could not produce the same therapeutic outcomes as in their nondiabetic counterparts* in vivo* [97]. Advanced glycation end products (AGEs) accumulate in the tissues of aged people, and these products are involved in diabetes and in musculoskeletal diseases. In 2005, Kume et al. [98] investigated the effect of AGEs on MSCs and showed that AGEs inhibited MSC proliferation, induced MSC apoptosis, and interfered with MSC differentiation into adipose tissue, cartilage, and bone. Another study examined type 2 diabetes-derived AD-MSCs and found that these cells had functional impairments in their multilineage potential and proliferative capacity because of prolonged exposure to high glucose concentrations [99]. We demonstrated that diabetic-derived AD-MSCs have an altered phenotype related to plasminogen activator inhibitor-1 (PAI-1) expression levels and display reduced fibrinolytic activity [100]. In this respect, our preliminary results and others suggest that the immunogenicity of MSCs could have related effects on the coagulation system [101, 102]. Thus, MSC-based therapy could lead to thrombotic events in particular recipients.

Although the possibility of healing with our own cells is extremely attractive, little is known regarding the influence of different disease states and concomitant medications on MSCs [103, 104]. Thus, although the use of autologous MSCs for cell therapy is widespread, their use in humans must be handled with extreme caution. Researching and analysing both the risks and benefits of this therapy in individual patients and for each disease state are necessary.

## 6. Safety and Efficacy in Clinical Trials

Several clinical trials are in progress to ensure the safety and efficacy of MSCs used as medicaments. For cell-based products, we must consider that cells are living products and that their interactions with body fluids remain unclear [100, 102, 105].

Phase I clinical trials are the first step in the investigation of a new drug and include pharmacokinetic and pharmacodynamic studies in which the patient's safety plays an essential role in the development of medicaments. The primary goal of phase II clinical trials is to provide preliminary information regarding the drug efficacy and safety supplement data obtained in phase I trials. Usually, safety evaluations are based on possible complications derived from the procedure in a time-dependent manner after the administration of the cells. Efficacy parameters focus on the improvement of clinical effects at a given time. MSC-based cell therapy is a relatively new therapeutic option for certain diseases, and data regarding the long-term monitoring of patients remain lacking. Nevertheless, the administration of MSCs is considered a feasible and safe procedure with no adverse events reported. However, the risks associated with stem cell therapy [106] must be considered because these risks increase the probability of the occurrence of an adverse event. The cell source, donor origin, product manufacturing, and recipient disease status are important factors related to the safety and efficacy of the use of MSCs. In this regard, the use of bovine proteins in the medium used to culture these cells [38] and the observed formation of ectopic tissue in animal models [107, 108], as well as malignant transformation [109, 110] and immune responses, must be evaluated before wider clinical applications and registration are accepted.

## 7. Clinical Manufacturing of MSC-Based Medicines

With the exception of haematopoietic stem cell transplants, stem cell therapies used for the treatment of any disease are considered drugs; therefore, their development, approval, and use must be in accordance with specific standards established for such medicines nationally and internationally. MSCs are called advanced therapy medicinal products (ATMPs) and are under regulation number 1394/2007. Relating production processes and development staff, clinicians, and researchers is obligatory to achieve GMP procedures under European regulations [111, 112]. Currently, no standardised manufacturing platform exists, although most facilities employ standard release criteria to measure sterility, viability, and chromosomal stability to meet European or FDA regulations [113]. Although regulation establishes common parameters to follow, different protocols are used to isolate these cells, and the processes, plating densities, and reagents used cause the results to differ from each other. Donor selection in terms of age and disease status is another variable to consider due to known MSC donor-to-donor heterogeneity [114]. The cell source is another important factor related to the efficacy of the product. As reported previously, MSCs derived from different tissues do not always achieve the same level of efficacy. Additionally, culture media used for the production of MSCs could affect the basic characteristics of cells; thus, designing a fully defined medium free of animal and human origins is crucial.

Thus far, no MSC-based medicine product has marketing authorisation in the European Union, although four gene and cell-based products have a valid marketing authorisation awarded by the European Medicines Agency. However, since 2011, three MSC products have received marketing approval in other regions [115] (Tables 2 and 3).

The MSC field continues its upward progression, with a growing number of established companies and ongoing clinical trials, but remaining challenges must be overcome. Bottlenecks exist regarding donor selection, cells sources, isolation protocols, culture media used, open-culture systems, bioreactors, and recipient disease status. Establishing a standardised and comparable process is also crucial to ensure biological and functional equivalence between product lots.

## 8. Concluding Remarks and Future Perspectives

Treatments based on the use of human stem cells are novel and promising therapeutic alternatives for some diseases. Spain is at the forefront of research using such treatments, and these treatments are developed and evaluated with great scientific rigor. Currently, the use of living cells as a medicinal product is becoming realistic. Cell therapy should be safe, pure, stable and efficient. Cell-based products are more complex and depend on the physiological and genetic heterogeneity of the patient. Obtaining as much information as possible with the tools we have at our disposal is essential for ensuring the safety, reliability, quality, and effectiveness of the manufactured product. MSCs are leading the way into a new era of regenerative medicine, and their multifaceted features make them powerful candidates to become tools to treat several diseases. However, their indiscriminate use has resulted in mixed outcomes in preclinical and clinical studies. While MSCs derived from diverse tissues share some common properties, they markedly differ in terms of their differentiation abilities, growth rates, healing capacity, and gene expression profile. Similarly, the disease statuses of donors and recipients are important factors to consider when using MSCs as therapeutic agents because factors such as the MSC behaviour with body fluids and specific disease environments remain unclear. Available data suggest that some tissue-specific MSCs are more appropriate than others according to particular pathologies. Equally, some evidence has indicated that certain patient profiles are not suitable to be treated with these therapies. Thus, multiple bottlenecks for the standardisation of therapeutic protocols exist. Future well-designed clinical trials and long-term monitoring of patients are crucial for obtaining additional information regarding the therapeutic use of MSCs.

## Figures and Tables

**Table 1:** Summary of mesenchymal stroma cell nomenclature.

Nomenclature	Year	Authors' references
CFU-F and osteogenic stem cells	1974	Friedenstein et al. [10]
Stromal stem cells	1988	Owen and Friedenstein [16]
Mesenchymal stem cells	1991	Caplan [17]
Mesenchymal progenitor cells	1999	Dennis et al. [18]
Skeletal stem cells	2000	Bianco and Robey [19]
MAPCs and mesodermal progenitor cells	2002	Jiang et al. [20]
Multipotent mesenchymal stromal cells	2006	Dominici et al. (ISCT) [21]
Medicinal signalling cells	2010	Caplan [22]

CFU-F: colony-forming unit fibroblasts; MAPCs: multipotent adult progenitor cells; ISCT: International Society for Cell Therapy.

**Table 2:** Advanced therapy medicinal products (ATMPs) with a valid marketing authorisation.

Trade name	Company	Authorised by	Cell type	Indication
Carticel^b^	Genzyme (Sanofi Biosurgery)	FDA (1997)	Autologous cultured chondrocytes	For the repair of symptomatic cartilage defects of the femoral condyle (medial, lateral, or trochlea) caused by acute or repetitive trauma in patients who have had an inadequate response to a prior arthroscopic or other surgical repair procedure

Chondron^d^	Sewon Cellontech Co., Ltd.	KFDA (2001)	Autologous chondrocyte implantation	Articular cartilage defects

Articell^d^	Duplogene	KFDA (2002)	Autologous cultured chondrocytes	Articular cartilage defects

Haloderm^d^	Tego Science	KFDA (2002)	Cultured epidermal autograft	Burn wounds

Kaloderma^d^	Tego Science	KFDA (2005)	Allogeneic keratinocyte sheet	Burn wounds

JACE^e^	J-TEC (Japan Tissue Engineering Co., Ltd.)	Japan's Ministry of Health, Labour & Welfare (2007)	Autologous cultured epidermis	Burn wounds

Chondrocelect^c^	Tigenix, Belgium	EMA (2009)	Autologous cultured chondrocytes	In adults to repair damage to the cartilage in the knee

Provenge^b^	Dendreon Corporation	FDA (2010) EMA (2013)	Autologous PB-MNS activated with FAP-GM-CSF (sipuleucel-T)	In autologous cellular immunotherapy for the treatment of asymptomatic or minimally symptomatic metastatic castration-resistant (hormone refractory) prostate cancer

LaViv^b^	Fibrocell Technologies, Inc.	FDA (2011)	Autologous cultured fibroblasts (azficel-T)	Indicated for the improvement of the appearance of moderate to severe nasolabial fold wrinkles in adults

Hemacord^b^	New York Blood Center, Inc.	FDA (2011)	Allogeneic HPC, cord blood	For use in unrelated donor haematopoietic progenitor cell transplantation procedures in conjunction with an appropriate preparative regimen for haematopoietic and immunological reconstitution in patients with disorders affecting the haematopoietic system that are inherited or acquired or that result from myeloablative treatment

Glybera^a^	UniQure Biopharma B.V., Netherlands	EMA (2012)	Alipogene tiparvovec	To treat adults with lipoprotein lipase deficiency who have severe or multiple attacks of pancreatitis (inflammation of the pancreas) despite maintaining a low-fat diet

Gintuit^c^	Organogenesis Incorporated	FDA (2012)	Allogeneic cultured keratinocytes and fibroblasts in bovine collagen	Allogeneic cellularized scaffold product indicated for topical (nonsubmerged) application to a surgically created vascular wound bed in the treatment of mucogingival conditions in adults

Ducord^b^	Duke University School of Medicine	FDA (2012)	Allogeneic HPC, cord blood	For use in unrelated donor haematopoietic progenitor cell transplantation procedures in conjunction with an appropriate preparative regimen for haematopoietic and immunological reconstitution in patients with disorders affecting the haematopoietic system that are inherited or acquired or that result from myeloablative treatment

JACC^e^	J-TEC (Japan Tissue Engineering Co., Ltd.)	Japan's Ministry of Health, Labour & Welfare (2012)	Autologous cultured cartilage	Articular cartilage defects

MACI^c^	Genzyme (Sanofi Biosurgery)	EMA (2013)	Matrix-induced autologous cultured chondrocytes	For implant to repair cartilage defects at the ends of the bones of the knee joint

Allocord^b^	SSM Cardinal Glennon Children's Medical Center	FDA (2013)	Allogeneic HPC, cord blood	For use in unrelated donor haematopoietic progenitor cell transplantation procedures in conjunction with an appropriate preparative regimen for haematopoietic and immunological reconstitution in patients with disorders affecting the haematopoietic system that are inherited or acquired or that result from myeloablative treatment

FAP-GM-CSF: prostatic acid phosphatase granulocyte-macrophage colony-stimulating factor; HPC: haematopoietic progenitor cells; PB-MNS: peripheral blood mononuclear cells.

^a^GTMP: gene therapy medicinal product; ^b^SCTMP: somatic cell therapy medicinal product; ^c^TEMP: tissue-engineered medicinal product; ^d^biotechnology-based product (KFDA Classification); ^e^medical devices (Japan Classification).

**Table 3:** MSC cell-based therapies with a valid marketing authorisation.

Trade name	Company	Authorised by	Cell type	Indication
Hearticellgram^*^	FCB PharmiCell	KFDA (2011)	Autologous BM-derived MSCs	Treatment for postacute myocardial infarction
Cartistem^*^	Medipost	KFDA (2012)	Allogeneic hUCB-MSCs	Treatment of traumatic and degenerative osteoarthritis
Prochymal^*^	Osiris Therapeutics Inc.	Health Canada (2012) New Zealand (2012)	Allogeneic BM-MSCs	Treatment of acute GvHD children who are unresponsive to steroids

MSCs: mesenchymal stem cells; BM: bone marrow; hUCB: human umbilical cord blood; GvHD: graft-versus-host disease; KFDA: Korean Food and Drug Administration.

^*^SCTMP: somatic cell therapy medicinal product.
